# Supplementary material for: The Gut Bacteria Dysbiosis Contributes to Chronic Graft-Versus-Host Disease Associated With a Treg/Th1 Ratio Imbalance
Source: Front Microbiol. 2022 Sep 8;13:813576. doi: 10.3389/fmicb.2022.813576 (PMC9493085; doi:10.3389/fmicb.2022.813576)
Supplement: Supplementary file 1 [file Data_Sheet_1.zip › P101SC18090073-01-B1-3-4_result/04.BetaDiversity/PCA/PCA12_with_cluster.pdf]

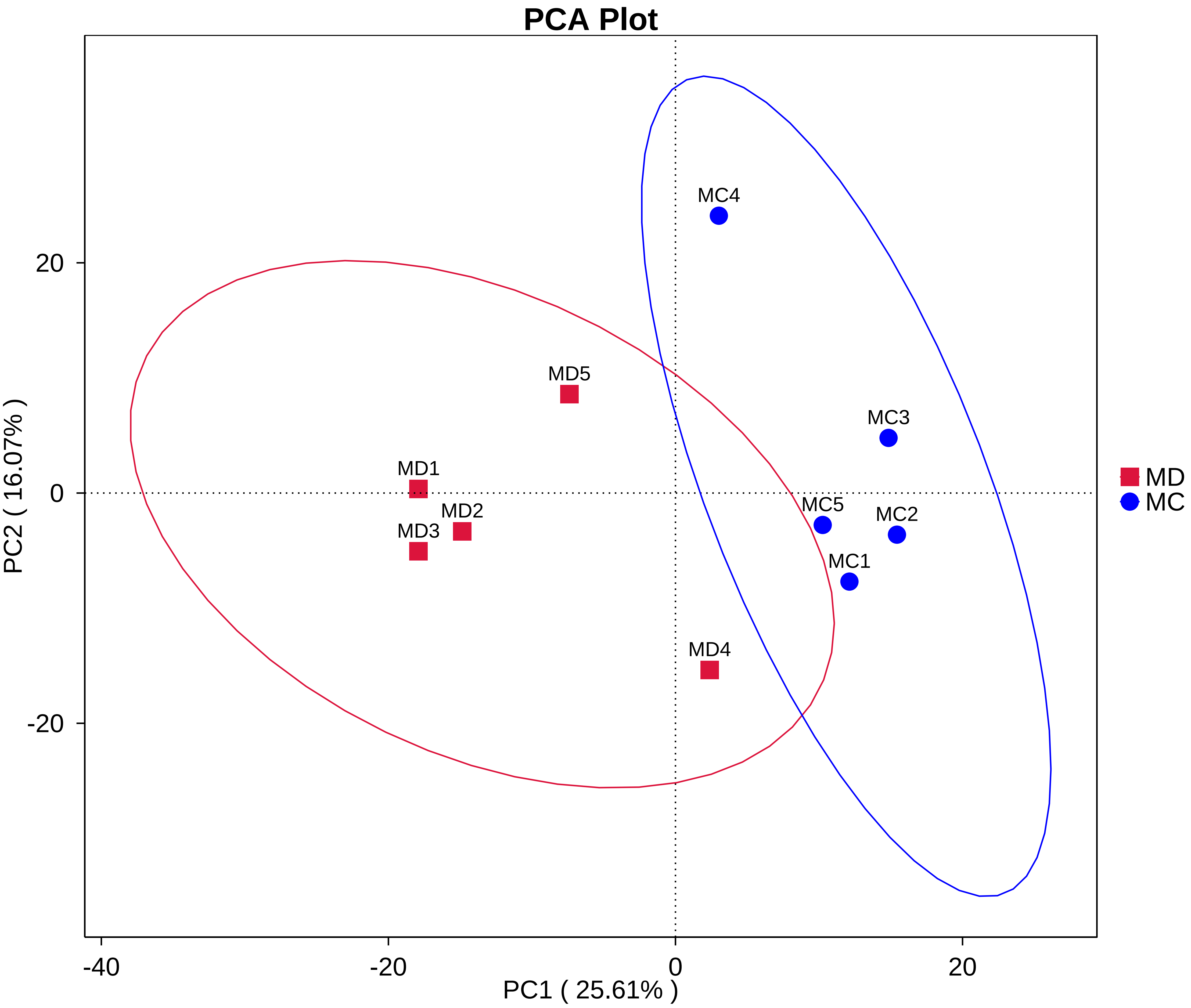

PCA Plot
MC4
MD5
MC3
MD1
MD2
MD3
MC5
MC2
MC1
MD4
MD
MC
20
0
-20
-40
-20
0
20
PC1 (25.61%)
PC2 (16.07%)
